# Docosahexaenoic Acid Modulates a HER2-Associated Lipogenic Phenotype, Induces Apoptosis, and Increases Trastuzumab Action in HER2-Overexpressing Breast Carcinoma Cells

**DOI:** 10.1155/2015/838652

**Published:** 2015-11-12

**Authors:** Graziela Rosa Ravacci, Maria Mitzi Brentani, Tharcisio Citrângulo Tortelli, Raquel Suzana M. M. Torrinhas, Jéssica Reis Santos, Angela Flávia Logullo, Dan Linetzky Waitzberg

**Affiliations:** ^1^Department of Gastroenterology, School of Medicine, University of São Paulo, LIM 35, Avenida Doutor Arnaldo 455, Cerqueira Cesar, 01246-903 São Paulo, SP, Brazil; ^2^Department of Radiology and Oncology, School of Medicine, University of São Paulo, São Paulo, SP, Brazil; ^3^Support Group for Research on Food and Nutrition (NAPAN), University of São Paulo, São Paulo, SP, Brazil; ^4^Cancer Institute of State of São Paulo (ICESP), São Paulo, SP, Brazil; ^5^Pathology Department, São Paulo Federal University (UNIFESP-EPM), São Paulo, SP, Brazil

## Abstract

In breast cancer, lipid metabolic alterations have been recognized as potential oncogenic stimuli that may promote malignancy. To investigate whether the oncogenic nature of lipogenesis closely depends on the overexpression of HER2 protooncogene, the normal breast cell line, HB4a, was transfected with HER2 cDNA to obtain HER2-overexpressing HB4aC5.2 cells. Both cell lines were treated with trastuzumab and docosahexaenoic acid. HER2 overexpression was accompanied by an increase in the expression of lipogenic genes involved in uptake (CD36), transport (FABP4), and storage (DGAT) of exogenous fatty acids (FA), as well as increased activation of “de novo” FA synthesis (FASN). We further investigate whether this lipogenesis reprogramming might be regulated by mTOR/PPAR*γ* pathway. Inhibition of the mTORC1 pathway markers, p70S6 K1, SREBP1, and LIPIN1, as well as an increase in DEPTOR expression (the main inhibitor of the mTOR) was detected in HB4aC5.2. Based on these results, a PPAR*γ* selective antagonist, GW9662, was used to treat both cells lines, and the lipogenic genes remained overexpressed in the HB4aC5.2 but not HB4a cells. DHA treatment inhibited all lipogenic genes (except for FABP4) in both cell lines yet only induced death in the HB4aC5.2 cells, mainly when associated with trastuzumab. Neither trastuzumab nor GW9662 alone was able to induce cell death. In conclusion, oncogenic transformation of breast cells by HER2 overexpression may require a reprogramming of lipogenic genetic that is independent of mTORC1 pathway and PPAR*γ* activity. This reprogramming was inhibited by DHA.

## 1. Introduction

Cell lipogenic metabolism has traditionally been considered a minor anabolic energy-storage pathway, yet its role in various cancers is increasingly being recognized [1–5]. Endogenous fatty acid (FA) biogenesis may constitute an oncogenic stimulus that drives normal epithelial cells towards malignancy [1–5]. Moreover, emerging evidence indicates that the oncogenic nature of human lipogenesis depends on the activity and/or expression of key protooncogenes, such as human epidermal growth factor receptor 2 (*HER2*) [1, 2, 5]. Amplification and overexpression of* HER2* are detected in approximately 20–30% of breast carcinomas and are associated with a poor prognosis [6–10]. Hyperactivation of HER2 promotes aberrant cell proliferation and tumorigenesis, thereby making HER2 an important therapeutic target against breast cancer [6–10].

Currently, the primary treatment for HER2-overexpressing tumors is trastuzumab (Herceptin) [11–14]. Trastuzumab is a monoclonal antibody that is designed to target the extracellular domain of HER2 and block its function. However, response rates for trastuzumab monotherapy have been reported to range from 12% to 34% with a median duration of 9 months [9, 10]. Thus, it appears that the mechanism of action of HER2 is not yet fully understood.

We previously showed that HER2 hyperactivation and signaling in breast cancer cells depend strongly on the location of the receptor within membrane lipid rafts [15]. In breast cancer cells, HER2 overexpression may be accompanied by an increase in cell membrane lipid raft microdomains, thereby establishing a vicious cycle of aberrant cell signaling [1, 15]. Recent experimental evidence revealed that the dimerization of HER2 (as a homo- or heterodimer with members of its own family) is associated with lipid rafts [1, 16]. In addition, HER2-mediated proliferation and survival signals depend on the colocalization of HER2 with other membrane proteins (e.g., integrins and extranuclear factor of the estrogen receptor [ER]) in lipid rafts [17, 18]. Accordingly, it is possible that an increase in the number of lipid rafts in HER2-overexpressing cells can enhance the activation of these oncogenic receptors [15].

To ensure lipid raft synthesis, HER2 promotes the activation of fatty acid synthase (FASN). Its final product, palmitate, is frequently used to synthesize membrane microdomains [1, 15, 19]. In a previous study, when this pathway was inhibited by omega-3 docosahexaenoic fatty acid (DHA), lipid rafts were disrupted and cell apoptosis was induced [15]. Thus, HER2 overexpression in breast cancer cells is associated with constitutive upregulation of the endogenous FASN-catalyzed biogenesis of palmitate. The upregulation of palmitate biogenesis represents a “lipogenic benefit” for the proliferation and survival of breast cancer cells by providing lipid raft components for the proper localization and activation of HER2 in the cell membrane [1, 2, 15, 19]. However, accumulation of palmitate in nonadipose tissue promptly stimulates lipolysis and apoptosis and can act as an inhibitory feedback signal for endogenous FA synthesis [1, 2, 20–22].

On the other hand, these events seem to be avoided in HER2-overexpressing breast carcinoma cells, through the conversion and storage of FAs as triglycerides by peroxisome proliferator-activated receptor gamma (PPAR*γ*) [1, 2]. Rather than preventing lipotoxicity, the transcriptional activation of PPAR*γ* increase the expression of genes related to uptake and transport of exogenous FA, contributing to the establishment of lipogenic phenotype in HER2-overexpressing cells [1, 2]. Therefore, in these cells, upregulation of FASN appears to be a downstream manifestation of an early and common deregulation of upstream regulatory circuits that affect the lipogenic genetic program [2]. It is believed that the regulation of lipogenesis occurs through mTOR protein [1, 2]. The HER2/mTOR pathway results in SREBP1 activation which can increase the transcription of PPAR*γ* endogenous ligands and regulates the expression of* FASN* [1, 2]. However, the details of this process remain unclear, since activation of components of the mTOR pathway, as mTORC1, may limit the survival signs by reducing Akt activity [1, 2]. Accordingly, it remains to be determined whether HER2 overexpression-mediated oncogenic transformation requires the activation of a genetic switch of lipogenic cell metabolism to maintain aberrant signaling that affects cell survival and proliferation.

From a molecular perspective, we hypothesized that the HER2 overexpression-mediated oncogenic transformation of breast cells involves a distinct lipogenic program that, in addition to FA synthesis, requires the coordinated expression of genes involved in the following: (a) the conversion and storage of excess FAs (e.g., palmitate) to triglycerides, thereby avoiding lipotoxicity; and (b) the uptake and transport of other exogenous FAs, which are necessary to maintain a constant supply of lipids/lipid precursors, membrane lipid raft production, and lipid-based posttranslational protein modifications in these highly proliferative cells. From a clinical perspective, the dependence of cancer cells on lipogenesis for survival and proliferation may represent the “Achilles' heel” of HER2-driven oncogenesis. Thus, lipogenic enzyme inhibitors, modulators of PPAR*γ* transcriptional activity, and, perhaps, dietary omega-3 polyunsaturated FAs (e.g., DHA) may provide novel therapeutic strategies for the clinical management of HER2-positive breast carcinomas and may increase the efficacy of standard therapies [2, 20, 21].

DHA is a potent PPAR*γ* regulator that has been shown to suppress adiposity in rodents and block adipogenesis in many adipocyte cell lines [23, 24]. As a modulator of cell membrane lipid composition, DHA can disrupt lipid rafts, thereby impairing HER2-regulated pathways and inducing cell apoptosis [15]. Therefore, our second hypothesis was that DHA could effectively modulate the lipogenic genetic switch associated with HER2 overexpression. In addition, we investigated whether DHA increases the trastuzumab action in HER2-overexpressing breast carcinoma cells.

## 2. Methods and Materials

### 2.1. Cell Culture

Parental, nontransformed HB4a cells and HER2-overexpressing HB4a variant cells, HB4aC5.2, were cultured in RPMI-1640 with 10% fetal bovine serum (FBS) (GIBCO, Invitrogen, Brazil) plus ampicillin, hydrocortisone, and insulin (Sigma-Aldrich, Brazil) at 37°C in a 10% CO_2_ humidified incubator [25, 26]. HB4a cells were derived from normal breast luminal cells. HB4aC5.2 cells were generated by cotransfecting HB4a cells with pJ5E.c-erbB-2, a plasmid containing the full-length normal human* HER2* cDNA, derived from the established breast cancer line BT474, under the control of the mouse mammary tumor virus-long terminal repeat (MMTV-LTR) promoter and SV40 polyadenylation signals [25, 26]. Five copies of pJ5E.c-erbB-2 were detected in the genome of the C5.2 clone, resulting in the expression of ~10^6^ HER2 receptors per HB4aC5.2 cell [25, 26]. Cells were tested periodically for mycoplasma (data not shown) and were authenticated by real-time reverse transcription polymerase chain reaction (RT-PCR) to evaluate* HER2* overexpression [15, 25, 26]. The HB4a and HB4aC5.2 cell lines were a generous gift from Michael J. O'Hare (Ludwig Institute for Cancer Research and University College London Breast Cancer Laboratory, Department of Surgery, London, UK).

### 2.2. Cell Treatments

Briefly, cells were seeded in flasks with medium containing 10% FBS and were allowed to adhere. After 24 h, the culture medium was replaced with fresh medium containing 10% FBS plus treatment agent. Cells were incubated for 72 h without changing the medium. Then, the cells were harvested with trypsin-EDTA (Sigma-Aldrich), and the viable cells were counted by Trypan Blue exclusion (Sigma-Aldrich) and a hemocytometer. Only samples with more than 95% viable cells were used.

#### 2.2.1. Treatment with the PPAR*γ* Inhibitor GW9662

The HB4a and HB4aC5.2 cell lines were treated with GW9662 diluted in dimethyl sulfoxide (DMSO) to 1 *μ*L/mL, on the basis of previous experimental studies using breast cancer cells [27]. The final concentration of DMSO did not exceed 0.1% in any case and was not cytotoxic in any of the cell lines tested at this concentration. GW9662 was kindly provided by Professor William Festuccia (University of Sao Paulo, Brazil).

#### 2.2.2. Treatment with Trastuzumab

The HB4a and HB4aC5.2 cell lines were treated with trastuzumab diluted in phosphate-buffered saline (PBS) to a concentration of 15 *μ*g/mL, on the basis of previous experimental and clinical studies [11–14]. Trastuzumab (Herceptin/Genentech, EUA) was kindly provided by Professor José Ernesto Belizário (University of Sao Paulo, Brazil).

#### 2.2.3. Treatment with DHA

DHA (C22:6n-3, Sigma-Aldrich) was dissolved in ethanol prior to emulsification in medium containing serum proteins. The final concentration of ethanol in the culture medium did not exceed 0.05%. This concentration was previously shown to be nontoxic to cells [28, 29]. The HB4a and HB4aC5.2 cell lines were treated with 100 *μ*M DHA for 72 h, based on previous testing of both cell lines with varying doses of DHA (25, 50, 75, and 100 *μ*M DHA for 24, 48, and 72 h in standard medium). Standard medium plus ethanol was used as a control.

### 2.3. Proliferation Experiments

To compare proliferation rates between the HB4a and HB4aC5.2 cell lines, 10^3^ cells were plated in triplicate and were allowed to attach to 96-well plates overnight in culture medium. The following day, the cells were washed with PBS and received fresh culture medium. After 5 d, the cells were harvested and combined with 0.5 mg/mL MTT. Four hours later, solubilization buffer was added and the cells were incubated for an additional 15 h. Spectrophotometry of the cells was then performed at 595 nm with a 655 nm reference filter. Calibration curves were established with a known number of cells, allowing the absorbance values to be converted into cell numbers.

To compare proliferation rates between treatments, cells were washed with PBS and received culture medium containing trastuzumab or GW9662 for 72 h. At several time points during this 72 h incubation (e.g., baseline and 12, 24, 48, and 72 h after treatment), a portion of the cells were harvested and combined with 0.5 mg/mL MTT to assess proliferation. Detection of proliferation was performed as described above.

### 2.4. RT-PCR

Total RNA was extracted from cells with the RNeasy Mini Kit (Qiagen, Brazil), in accordance with the manufacturer's instructions. RNA concentration and purity were determined with a spectrophotometer (NanoDrop ND-1000 UV-Vis Spectrophotometer, NanoDrop Technologies) by calculating the ratio of optical density at wavelengths of 260 nm and 280 nm. The cDNA was synthesized by reverse transcription from 2 *μ*g of RNA with the Superscript III Reverse Transcriptase Kit (Invitrogen, Brazil), according to the manufacturer's instructions.

Each PCR sample included 2.5 *μ*L of cDNA, 5 *μ*L of SYBR Green I (Molecular Probes), 1.1 *μ*L of MgCl_2_, 0.9 *μ*L of H_2_O DEPC, and 0.5 *μ*L of primers specific for the gene of interest (Table 1). PCR samples were amplified using a Rotor Gene 3000 System. After each run, the melting curve was analyzed to assess the reaction specificity.

### 2.5. Western Blotting

The HB4a and HB4aC5.2 cell lines (10^5^ cells/25 cm^2^ flask) were treated with 5 mL of medium containing 10% FBS, with or without DHA or trastuzumab. After 72 h, the cells were pelleted and proteins were extracted in a lysis buffer containing 50 mM Tris-HCl (pH 7.4), 150 mM NaCl, 1% NP-40, 0.5% sodium deoxycholate, 0.1% sodium dodecyl sulfate (SDS), and protease inhibitors. Protein concentrations were determined with the Bradford assay. Cell lysates (40 *μ*g) were boiled for 5 min in Laemmli buffer before being loaded on 10% acrylamide gels for SDS-polyacrylamide gel electrophoresis. Separated proteins were transferred to nitrocellulose membranes. The FASN protein was separated on a 6% acrylamide gel. Membranes were blocked for 1 h in Tris-buffered saline containing 0.05% Tween-20 and 5% skim milk before being incubated with primary antibodies (Cell Signaling Technology, USA, 1 : 1000). After 16 h at 4°C, membranes were incubated with anti-rabbit IgG antibodies (1 : 5000). Bound antibodies were visualized by enhanced chemiluminescence reagent (GE). Membranes were subjected to autoradiography, and quantitative densitometric analysis was performed with the Scion Image software package.

### 2.6. Flow Cytometry Analysis of Cell Death

The HB4a and HB4aC5.2 cell lines (10^5^ cells/25 cm^2^ flask) were treated with 5 mL of medium containing 10% FBS, with DHA, trastuzumab, GW9662, or DHA plus trastuzumab, for 72 h. Treated cells were fixed in 70% ethanol at −20°C and stained for 30 min at room temperature with 20 *μ*g/mL propidium iodide (PI) (Sigma-Aldrich), Triton-X (0.1% v/v), and 200 *μ*g/mL DNase-free RNase diluted in PBS. Cells from each sample were analyzed for DNA content with a Becton Dickinson FACS Caliber instrument. Percentages of cells in the sub-G1, G0/G1, and S/G2/M phases of the cell cycle were determined with the Cell Quest software package. Cell death was measured according to the percentage of cells in the sub-G1 region of the fluorescence scale that contained hypodiploid DNA.

### 2.7. Statistical Analysis

Data are presented as mean ± standard error of the mean (SEM) of three independent experiments performed for each variable. For both cell lines, relative gene expression was expressed as the ratio between target gene expression and the mean expression of the constituent genes (i.e.,* UBC*,* HMBS*, and *β-actin*, selected because they did not present significant variations in expression between the untreated and treated cell lines). For comparisons between cell lines, gene expression values obtained by real-time PCR were normalized to the results obtained from the HB4a cell line. For comparisons between treatments, gene expression values obtained by real-time PCR were normalized to results obtained from cell lines treated with ethanol, DMSO, or PBS (controls [CNTs]). Data were obtained from experiments performed in quadruplicate (or duplicate, for real-time PCR assays). The statistical significance of differences was assessed by one-way analysis of variance (ANOVA), followed by the Bonferroni post-test. The significance level was set at *P* ≤ 0.05.

## 3. Results

### 3.1. In HB4aC5.2 Cells, HER2 Overexpression Is Associated with Activation of a Lipogenic Genetic Switch

**Figure 1:**
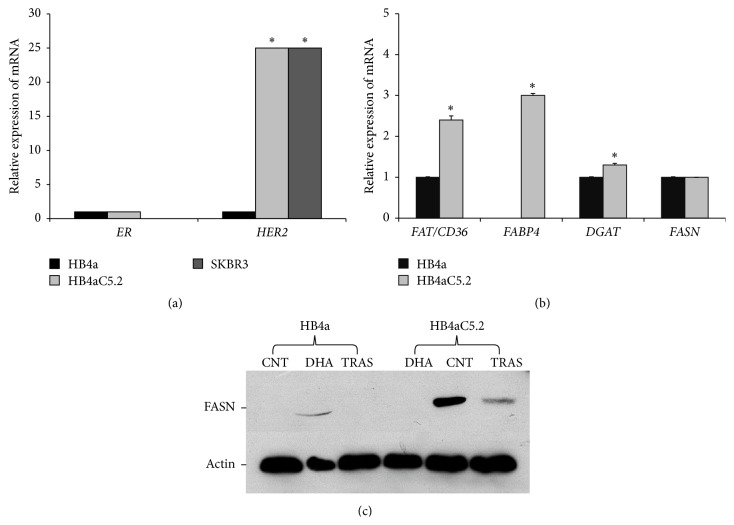
HER2 overexpression and activation of a lipogenic genetic program. (a-b) Relative expression levels of* HER2* and* ER* mRNAs in the HB4a, HB4aC5.2, and SKBR3 cell lines (a) and levels of* FAT/CD36*,* FABP4*,* DGAT*, and* FASN* mRNAs in the HB4a and HB4aC5.2 cell lines (b). The experiment was performed in quadruplicate. The PCR reaction was performed in duplicate. ^*∗*^ *P* < 0.001 versus HB4a. (c) Activation of FASN protein in HB4a and HB4aC5.2 cells. Immunoprecipitated proteins were subjected to Western blotting for FASN and *β*-actin, as controls. CNT: control cells; DHA: cells treated with 100 *μ*M DHA for 72 h; TRAS: cells treated with 15 *μ*g/mL trastuzumab for 72 h.

To test the hypothesis that HER2 overexpression requires activation of a lipogenic genetic program for oncogenic transformation, the immortalized human mammary luminal epithelial cell line, HB4a, was transfected with* HER2* cDNA to generate the HB4aC5.2 cell line. Oncogenic transformation was assessed by RT-PCR. The HB4aC5.2 cell line expressed* HER2* mRNA at levels equivalent to the tumor-derived cell line, SKBR3, but should be identical to the HB4a cell line in all other aspects (including* ER* mRNA levels) (Figure 1(a)). The ER-negative SKBR3 cell line is characterized by* HER2* amplification.

Real-time PCR analysis detected increased expression of lipogenic genes related to FA uptake (fatty acid translocase gene/cluster of differentiation 36,* FAT/CD36*), FA transport (fatty acid binding protein 4,* FABP4*), and lipid storage (diacylglycerol acyltransferase,* DGAT*) in HB4aC5.2 cells compared to HB4a cells (Figure 1(b)). Interestingly,* FASN* expression was not altered by HER2 overexpression (Figure 1(b)). However, according to Western blot analysis, HB4aC5.2 cells exhibited increased activation of the FASN protein compared to HB4a cells (Figure 1(c)).

### 3.2. DEPTOR, but Not mTOR/PPAR*γ*, May Be Associated with Activation of a Lipogenic Genetic Program in HB4aC5.2 Cells

**Figure 2:**
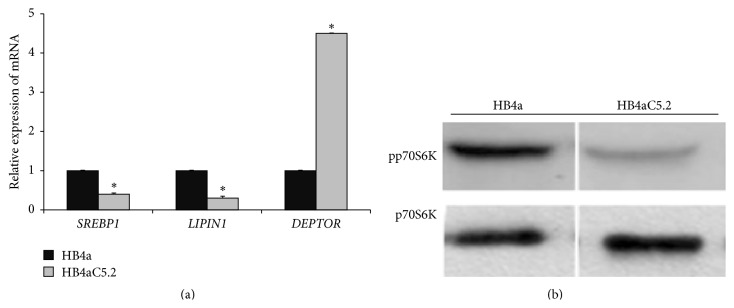
Relative expression of mTORC1 pathway markers. (a) Relative expression of* SREBP1*,* LIPIN1*, and* DEPTOR* in HB4a and HB4aC5.2 lines. Data were obtained from an experiment performed in quadruplicate. The real-time PCR reaction was performed in duplicate. ^*∗*^ *P* < 0.001 versus HB4a. (b) Detection of phosphorylated and nonphosphorylated forms of p70S6K (pp70S6K and p70S6K, resp.) by Western blot in HB4a and HB4aC5.2 cells.

Activation of mTOR pathway components, mainly complex 1 (mTORC1) and the p70S6K1 (p70 ribosomal S6 kinase 1) protein, and expression of* SREBP1* (sterol regulatory element-binding protein 1) and* LIPIN1* may contribute to lipogenesis by promoting the production of endogenous ligands for PPAR*γ* [2, 30, 31]. Unexpectedly, both RT-PCR (Figure 2(a)) and Western blotting (Figure 2(b)) assays showed that all of these mTORC1 pathway markers were decreased in HB4aC5.2 cells, but not in HB4a cells (Figure 2).

**Figure 3:**
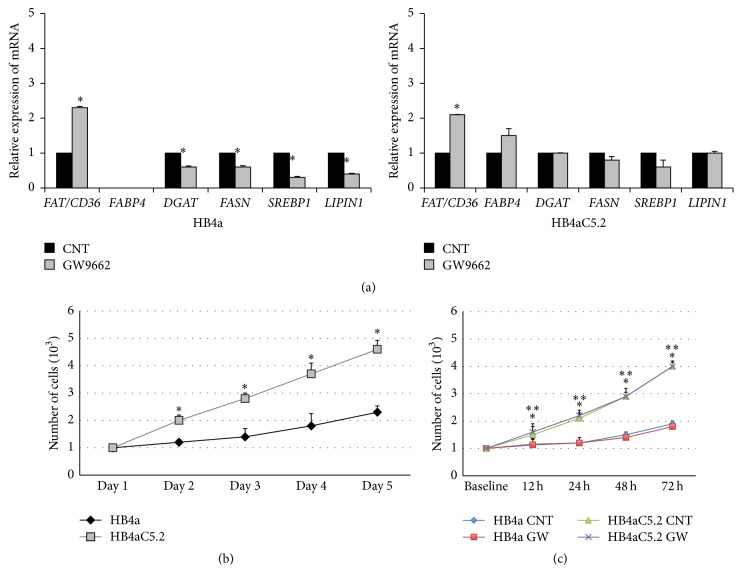
Effects of the PPAR*γ* inhibitor GW9662 on HB4a and Hb4aC5.2 cells. (a) Relative expression of* FAT/CD36*,* FABP4*,* DGAT*,* SREBP1*,* LIPIN1*, and* FASN* in HB4a and HB4aC5.2 cells treated with GW9662 (1 *μ*L/mL) for 72 h. DMSO was used as a control (CNT). Data were obtained from an experiment performed in quadruplicate. The real-time PCR reaction was performed in duplicate. ^*∗*^ *P* < 0.001 versus CNT. Proliferation rates for untreated (b) and treated (c) HB4a and HB4aC5.2 cells. Untreated cells were cultured in standard medium without additional stimulation for 5 d. Treated cells were incubated with medium containing GW9662 (GW) or DMSO as a control (CNT) for 0, 12, 24, 48, and 72 h. (b) ^*∗*^ *P* < 0.001 versus HB4a. ^*∗∗*^ *P* < 0.001 versus HB4a CNT. (c) ^*∗*^ *P* < 0.001 versus HB4aC5.2 CNT. ^*∗∗*^ *P* < 0.001 versus HB4aC5.2 GW.

When HB4a cells were treated with a PPAR*γ* selective antagonist (GW9662) and RT-PCR assays were performed, all of the PPAR*γ*-target regulatory genes via mTORC1 were decreased (with the exception of* FAT/CD36*, which showed increased expression) (Figure 3). Intriguingly, when HB4aC5.2 cells were treated with GW9662, expression levels of* FAT/CD36* increased, but the expression levels of the other genes remained unchanged (Figure 3).

Consistent with these findings, an increase in expression of* DEPTOR* (the main inhibitor of the mTOR pathway) was detected in HB4aC5.2 cells, but not in HB4a cells (Figure 2(a)). When overexpressed,* DEPTOR* can inhibit the activation of both complexes of the mTOR pathway, but especially mTORC1 [32–35]. The observed downregulation of* SREBP1* (Figure 2(a)) and its regulatory protein p70S6K1 (Figure 2(b)) confirmed the inhibition of mTORC1 activation and supported the possibility that DEPTOR activity was increased in HB4aC5.2 cells.

Although an association between activation of the mTORC1 pathway and cell proliferation has been observed in several cancers, this relationship was not observed in the present study [31, 32, 36]. Instead, the hyperproliferative phenotype induced by HER2 in HB4aC5.2 cells appeared to be independent of this pathway. The increased rate of proliferation of HB4aC5.2 cells compared to HB4a cells was independent of additional mitogen stimulation (Figure 3(b)). Treatment with GW9662 did not affect the proliferation rate of either cell line (Figure 3(c)).

### 3.3. In HB4aC5.2 Cells, Trastuzumab Treatment Was Accompanied by Increased FASN Gene Expression and Decreased FASN Protein Activation, While the Cell Proliferation Rate Remained Unchanged

**Figure 4:**
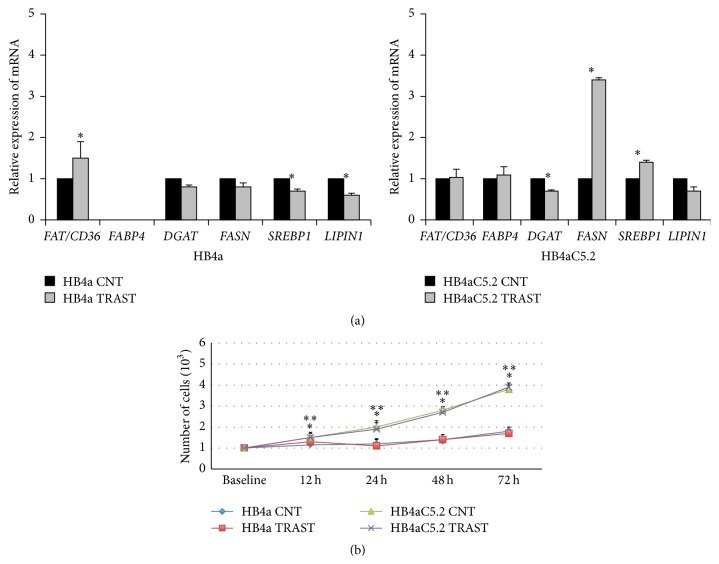
Effect of the HER2 inhibitor trastuzumab on HB4a and Hb4aC5.2 cells. (a) Relative expression of* FAT/CD36*,* FABP4*,* DGAT*,* FASN*,* SREBP1*, and* LIPIN1* in HB4a and HB4aC5.2 cells treated with trastuzumab (Herceptin, 15 *μ*g/mL for 72 h) or PBS as a control (CNT). Data were obtained from an experiment performed in quadruplicate. The real-time PCR reaction was performed in duplicate. ^*∗*^ *P* < 0.001 versus CNT. (b) Proliferation rates of HB4a and HB4aC5.2 cells treated with trastuzumab (TRAST) or PBS as a control (CNT) for 0, 12, 24, 48, and 72 h. ^*∗*^ *P* < 0.001 versus HB4aC5.2 CNT. ^*∗∗*^ *P* < 0.001 versus HB4aC5.2 TRAST.

Real-time PCR analyses of* FAT/CD36*,* FABP4*,* DGAT*,* FASN*,* SREBP1*, and* LIPIN1* revealed that trastuzumab treatment was associated with a decrease in* DGAT* mRNA and increases in* SREBP1* and* FASN* mRNA levels in HB4aC5.2 cells (Figure 4(b)). However, these results were not observed in HB4a cells (Figure 4(a)). Moreover, despite the significant increase in* FASN* transcription that was induced by trastuzumab treatment in HB4aC5.2 cells, activation of the FASN protein was inhibited in this cell line (Figure 1(c)).

In HB4aC5.2 cells, trastuzumab treatment did not influence cell proliferation, as detected by MTT assays (Figure 4(b)).

### 3.4. In HB4aC5.2 Cells, DHA Treatment Affected the Activation of a Lipogenic Genetic Program to Induce Cell Death and Improve Trastuzumab Action in Parallel with a Decrease in DEPTOR Transcription

**Figure 5:**
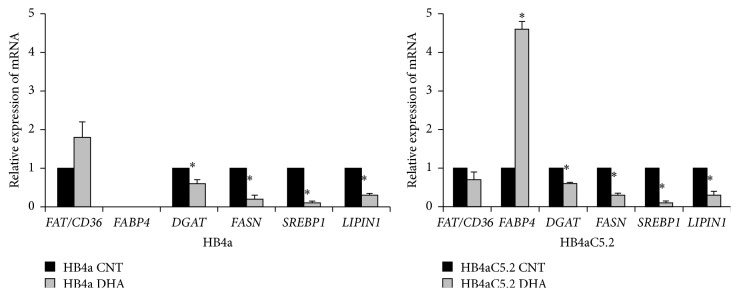
Effect of DHA on HB4a and Hb4aC5.2 cells. Relative expression of* FAT/CD36*,* FABP4*,* DGAT*,* FASN*,* SREBP1*, and* LIPIN1* in HB4a and HB4aC5.2 cells treated with DHA (100 *μ*M for 72 h) or ethanol as a control (CNT). Data were obtained from an experiment performed in quadruplicate. The real-time PCR reaction was performed in duplicate. ^*∗*^ *P* < 0.001 versus CNT.

**Figure 6:**
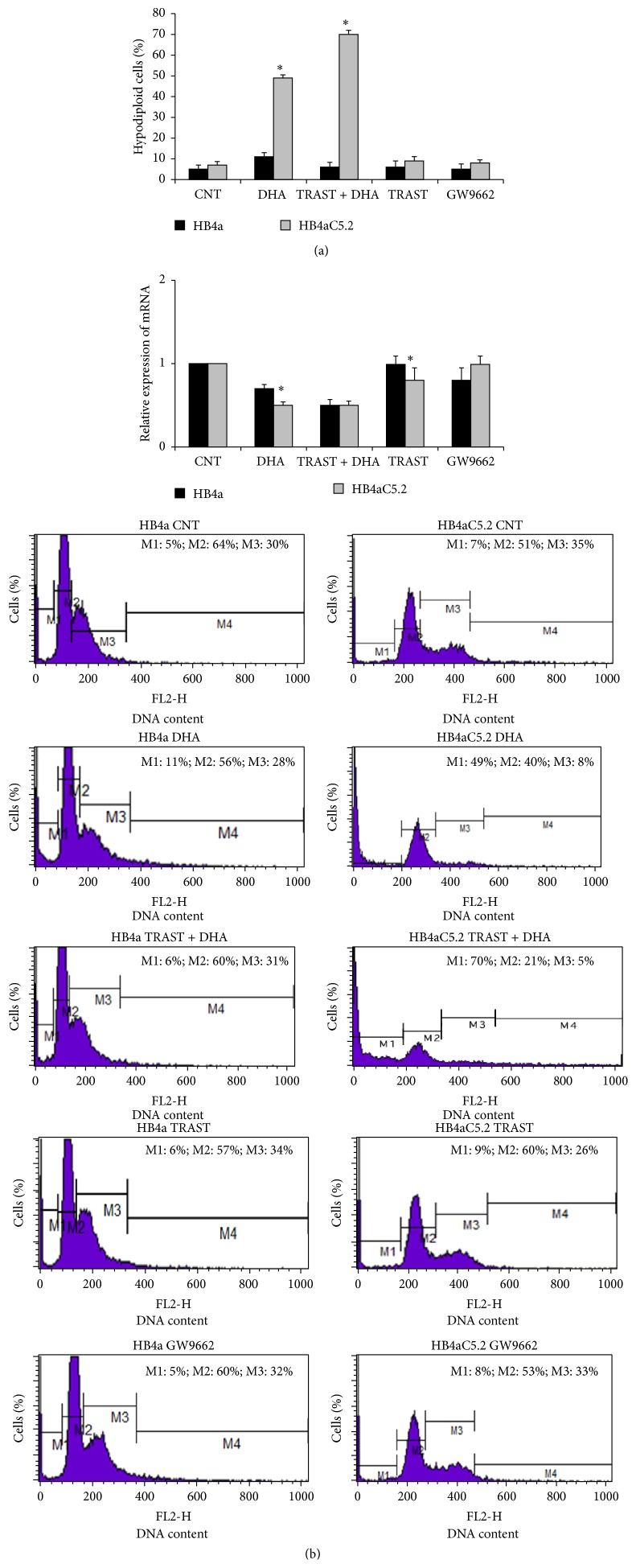
Percentage of cell death induced by different treatments and relative expression of* DEPTOR*. (a) Percentage of cell death induced by 100 *μ*M DHA, 15 *μ*g/mL trastuzumab (TRAST), 15 *μ*g/mL trastuzumab + 100 *μ*M DHA, and 1 *μ*L/mL GW9662 for 72 h in HB4a and HB4aC5.2 cells. Culture medium was used as a control (CNT). Subsequently, all cells were stained with PI and analyzed by flow cytometry. Cells exhibiting hypodiploid DNA content were considered to have undergone cell death. ^*∗*^ *P* < 0.001 versus HB4a. ^*∗*^ *P* < 0.001 DHA versus CNT, TRAST + DHA, TRAST, and GW9662. Data were obtained from an experiment performed in triplicate. (b) Relative expression of* DEPTOR* gene in HB4a and HB4aC5.2 cells treated with 100 *μ*M DHA, 15 *μ*g/mL trastuzumab (TRAST), 100 *μ*M DHA plus 15 *μ*g/mL trastuzumab (TRAST + DHA), 1 *μ*L/mL GW9662, or culture medium as a control (CNT) for 72 h. Data were obtained from an experiment performed in quadruplicate. The real-time PCR reaction was performed in duplicate. ^*∗*^ *P* < 0.001 versus CNT. Results from PBS and DMSO treatments (vehicle dilution) did not differ from those obtained from treatment with standard culture medium. The histograms below the graphs represent the data obtained by flow cytometry. M1: percentage of hypodiploid cells (e.g., cell death); M2: percentage of cells in the G0/G1 phase of the cell cycle; M3: percentage of cells in the S/G2/M phase of the cell cycle; and M4: cell debris.

Previous studies have shown that DHA exhibits a triacylglycerol-lowering effect* in vitro* and* in vivo* and reduces the expression levels of lipogenic genes [37, 38]. However, the mechanisms responsible for these effects remain unknown. Real-time PCR analyses of* FAT/CD36*,* FABP4*,* DGAT*,* FASN*,* SREBP1*, and* LIPIN1* were performed for HB4aC5.2 and HB4a cells, with or without DHA treatment. DHA reduced the expression levels of all of the genes assayed, except* FABP4*, and inhibited the activation of FASN in HB4aC5.2 cells (Figure 5). Although both cell lines achieved very similar expression levels for the genes (except* FABP4*), DHA treatment induced cell death only in HB4aC5.2 cells (Figure 6(a)). Neither trastuzumab nor GW9662 alone affected the rate of cell death for either cell line (Figure 6(a)); however, combined treatment with DHA and trastuzumab increased cell death in the HB4aC5.2 cells when compared with DHA and trastuzumab alone (Figure 6(a)).

Finally, the relative expression of* DEPTOR* in the HB4a and HB4aC5.2 cells treated with DHA, trastuzumab, DHA plus trastuzumab, GW9662, or culture medium (as a control) was detected by real-time PCR. In HB4aC5.2 cells, DHA-induced toxicity was accompanied by a decrease in* DEPTOR* transcription. A greater decrease in* DEPTOR* transcription was induced by DHA plus trastuzumab treatment compared to treatment with DHA alone (Figure 6(b)).

## 4. Discussion

We hypothesized that the HER2 overexpression-mediated oncogenic transformation of breast cells involves a distinct lipogenic program that, in addition to FA synthesis, requires the coordinated expression of genes involved in the following: (a) the conversion and storage of excess FAs to triglycerides, thereby avoiding lipotoxicity; and (b) the uptake and transport of other exogenous FAs, which are necessary to maintain a constant supply of lipids/lipid precursors, in these highly proliferative cells.

For a model, we chose a transformed, immortalized cell line with a strictly luminal phenotype that has been specifically engineered to overexpress HER2 (HB4aC5.2) but is identical to its parental strain (HB4a) in all other aspects. This permits a cleaner analysis of the specific effects of enhanced HER2 levels on luminal epithelial cell function and phenotype, unlike most tumor cell lines already established and described in* in vitro* studies, which show multiple genetic aberrations other than overexpression of HER2 receptors. By using HB4aC5.2 and HB4a cells, we were able to analyze the specific effects that enhancing HER2 levels had on the lipogenic phenotype [25, 26].

Elevated levels of HER2 expression have been observed in human breast cancers, with levels of* HER2* amplification ranging from 2-fold to greater than 20-fold [25, 26, 39, 40]. One consequence of HER2 overexpression in epithelial cells is hyperproliferation [25, 26], which requires an increase in FA synthesis in order to provide building materials for new membranes and lipid rafts [1–3, 15]. This requirement to lipogenesis for survival and proliferation may represent a target treatment in HER2-driven oncogenesis [1–3, 15]. Experimental and clinical studies have shown that the early stages of tumorigenesis in HER2-overexpressing breast cancer cells are associated with increased activation of the FASN-mediated synthesis of palmitic acid, which is often used to form lipid rafts [1–3, 5, 15, 19, 41, 42]. In the present study, HER2 overexpression was accompanied by an increase in FASN protein activation, in parallel with the increased expression of* DGAT*, a gene that encodes an enzyme involved in the final step of triglyceride synthesis [43]. Thus, breast cancer cells that overexpress HER2 and have increased FASN activity may sustain their proliferation and avoid lipotoxicity by converting and storing excess palmitate as triglycerides.

The increase in* DGAT* expression was accompanied by an increase in expression levels of* FAT/CD36* and* FABP4*, which encode proteins involved in the uptake and transport of FAs. These processes facilitate the synthesis of cellular membranes during cell proliferation suggesting that HER2-overexpressing breast cancer cells alter their metabolism to improve triglyceride synthesis and lipid uptake/incorporation for cell proliferation [1–3]. These findings suggest that a lipogenic phenotype is required and possibly induced by HER2 overexpression and the observed increase in FASN activation comprises only one part of a much larger lipogenic program in such cells.

Although the exact mechanism linking HER2 signaling with lipogenesis remains unknown, accumulating evidence indicates that activation of PPAR*γ* via the mTOR pathway may regulate this process [1, 2, 31, 33–35]. According to our results, the mTORC1 activity (component of the mTOR pathway) in HB4aC5.2 cells was low suggesting that PPAR*γ* may be regulated through a pathway other than mTOR. Indeed, this lipogenic program required for oncogenic transformation in HB4aC5.2 cells was not found to be coordinated by PPAR*γ* activity because the blockage of its activity did not alter the expression of the lipogenic genes assayed in these cells. This finding raises the possibility that HER2 overexpression may employ another mechanism to maintain or generate the lipogenic phenotype [33, 34]. Based on the present findings,* DEPTOR* may be a potential mediator.

*DEPTOR* is the main inhibitor of the mTOR pathway and may regulate the lipogenesis process [33, 34]. In adipocytes,* DEPTOR* expression promotes adipogenesis, whereas inhibition of* DEPTOR* blocks this process [33]. In animal models,* DEPTOR* overexpression is responsible for the accumulation of white adipose tissue, and in humans, it is associated with some degree of obesity [33].

Experimental evidence suggests that* DEPTOR* is a potent activator of Akt-mediated survival pathways [33, 34]. For example, when* DEPTOR* is overexpressed, mTORC1 activity is reduced and the PI3K/mTORC2/Akt pathway is activated via release of the inhibitory feedback that mTORC1 imposes on mTORC2 [32]. Interestingly, this indirect mode of Akt protein activation appears to be important for the viability of thyroid carcinoma and multiple myeloma cells [35].* DEPTOR* overexpression and reduced mTORC1 complex activity have been detected in approximately 28% of patients with multiple myeloma, and these patients had lower survival rates [35]. Experimentally,* DEPTOR* overexpression has been shown to reduce protein synthesis and cell growth in multiple myeloma cells, while activating survival signals from PI3K/Akt proteins.* DEPTOR* downregulation has also been shown to promote cell death [35].

We previously reported that HER2 overexpression in HB4aC5.2 cells is accompanied by hyperactivation of Akt [15]. In the present study, a significant increase in* DEPTOR* expression and decreases in the expression levels of mTORC1 pathway members (i.e., p70S6K and* SREBP1*) were observed in HB4aC5.2 cells. It may be that the increase in* DEPTOR* expression provides an important oncogenic advantage for HB4aC5.2 cells.* DEPTOR* may help regulate lipogenesis to facilitate proliferation and may enhance Akt activation in favor of cell survival. Akt can promote cell survival by various mechanisms, including regulation of transcription factors other than PPAR*γ* [44, 45]. As an Akt activator [35],* DEPTOR* may enhance the expression of lipogenic genes that are targeted not only by PPAR*γ* but also by other transcription factors. This explanation would account for the observed increase in lipogenic gene expression in HER2-overexpressing cells while PPAR*γ* activity was not detected.

Overall, the present results show that the oncogenic transformation of HB4aC5.2 cells by* HER2* overexpression appears to promote a lipogenic environment conducive to cell proliferation and cell survival. Furthermore, this environment may be potentially dependent on* DEPTOR* overexpression. Accordingly, inhibitors of lipogenic enzymes, modulators of* DEPTOR* gene expression, and FA supply could impair HER2-mediated oncogenesis.

Several reports have indicated that omega-3 polyunsaturated FAs, such as DHA, can act as efficient anti-HER2 therapeutics [2, 15, 46]. Previous studies have largely attributed the DHA sensitivity of HER2-positive cells to the ability of this FA to suppress HER2 expression or HER2-mediated pathways by different mechanisms (e.g., lipid raft disruption). However, it is possible that the supplementation of highly lipogenic HER2-positive cells with FAs other than palmitate could trigger the generation of reactive oxygen species and cell death [1, 2, 15, 46]. In the present study, DHA treatment of the HB4aC5.2 cells led to a decrease in* DGAT* expression and an induction of apoptosis. These findings indicate the diminished ability of these cells to mediate the nontoxic accumulation of lipids in their triglyceride form.

Interestingly, the increased expression levels of* FAT/CD36* and* FABP4* that were observed in HB4aC5.2 cells were maintained after DHA treatment of these cells. It is possible that these cells were able to capture and transport DHA for cell membrane formation. Such preferential use of DHA would inhibit cell survival and proliferation, due to alterations in the formation of cell membrane lipid rafts [47–49]. Previously, we reported that DHA treatment of HB4aC5.2 cells increased DHA and decreased palmitic acid percentages in cell membranes [15]. These changes were observed concomitantly with a decrease in the number of lipid rafts, which, in turn, may have impaired HER2-mediated signaling [15]. This possibility was supported by the simultaneous decrease in activation of the Akt and ERK1/2 proteins [15]. In the present study, DHA treatment led to decreased activity of the FASN protein and lower expression levels of* DGAT* and* DEPTOR*. Besides, the DHA treatment plus trastuzumab was able to increase cell death percentage, in HB4aC5.2 cells, when compared with DHA and trastuzumab alone.

Taken together, these data support the possibility that the mechanism responsible for DHA-related toxicity in HB4aC5.2 cells includes a disturbance of the lipogenic genetic program. This disturbance may be mediated, at least in part, by* DEPTOR* and is distinct from trastuzumab, which seems to disturb FASN activity by different mechanisms. The cytotoxic pathways that are involved seem to be complementary, to improve cell death only in HER2-overexpressing HB4aC5.2 cells. Moreover, DHA appears to increase the sensitivity of cells to death by modulating a HER2-driven lipogenic genetic program. These findings support the use of DHA as a candidate therapeutic agent for minimizing HER2-mediated oncogenesis in breast cancer cells by disturbing a PPAR*γ*-independent lipogenic phenotype associated with* HER2* overexpression.

One limitation of the present study design is its specificity. For example, the HB4aC5.2 cell line was designed to overexpress HER2. These cells exhibited noninvasive and proliferative characteristics and expressed luminal epithelial markers. Consequently, the present results should be analyzed with caution when extrapolated to more complex models or other types of cells representing different breast cancer tumor stages. Additionally, the results provided in this study are valid only for DHA, which may have distinct or different antitumoral effects than other omega-3 fatty acids, such as eicosapentaenoic acid (EPA), and omega-6 fatty acids, such as arachidonic acid (AA) [50]. Indeed, it is widely recognized that DHA reduce and AA increase the risk of breast cancer in experimental and clinical studies [50–53]. However, it is noteworthy that the opposite effects between these FA also seem to extend to the cellular lipogenesis [23, 24, 54, 55]. Some authors have shown that DHA suppress adiposity in rodents and block adipogenesis in many adipocyte cell lines [23, 24], while AA is associated with increase of adipogenesis [54, 55]. According to our results, the DHA antitumoral effect was accompanied by cellular lipogenesis decrease. In this scenario, an important question emerges: Is it possible that AA effect toward tumorigenesis [50–53] might be associated with its capacity to increase cellular lipogenesis [54, 55]? Moreover, are tumor lipogenic phenotype and FA (DHA and AA) effects associated? From a clinical perspective, considering that dietary fat is part of modifiable risk of breast cancer, further studies should be conducted to evaluate the role of different FA in lipogenesis and breast cancer progression.

Together, our data demonstrate that an oncogenic transformation of* HER2*-expressing breast cancer cells supercharges cell lipogenesis via coexpression of various genes involved in the synthesis, uptake, transport, and storage of FAs. DHA treatment disturbs this lipogenic state by inducing cell death and increasing the action of trastuzumab. Therefore, DHA may represent a useful tool for controlling the aberrant signaling triggered by HER2. Nutritional interventions may constitute a new approach for improving conventional therapies, without adversely affecting patient quality of life. In particular, DHA supplementation in combination with other drugs, such as inhibitors of HER2 (trastuzumab), should be explored as a treatment strategy for breast cancer.

## Figures and Tables

**Table 1:** Primers used for RT-PCR.

GENE	Forward (5′-3′)	Reverse (5′-3′)
*HER2/neu*	GGGCTGGCCCGATGTATTTGAT	ATAGAGGTTGTCGAAGGCTGGGC
*FAT/CD36*	TGCAAAACGGCTGCAGGTCA	TGGTTTGTGCTTGAGCCAGGTTTAT
*FABP4*	GGAGTGGGCTTTGCCACCAGG	CGCCTTTCATGACGCATTCCACC
*DGAT*	TCGCCTGCAGGATTCTTTAT	GCATCACCACACACCAGTTC
*DEPTOR*	GCGGAGCTGCCCCGAACAAA	GTGCAGCCTGAGCCGTAGCTG
*SREBP1*	ACAGTGACTTCCCTCGCCTAT	GCATGGACGGCTACATCTTCAA
*FASN*	CCGAGACACTCGTGGGCTA	CTTCAGCAGGACATTGATGCC
*UBC*	ACCCAAGAAAAGCACAAGG	AGCCCAGTGTTACCACCAAG
*HMBS*	CAAAGATGAGAGTGATTCGC	CACACTGTCCGTCTGTATGC
*β-actin*	GGGACGACATGGAGAAAATC	GGGTGTTGAAGGTCTCAAAC
